# WSG, a Glucose-Rich Polysaccharide from *Ganoderma lucidum*, Combined with Cisplatin Potentiates Inhibition of Lung Cancer *In Vitro* and *In Vivo*

**DOI:** 10.3390/polym13244353

**Published:** 2021-12-13

**Authors:** Wei-Lun Qiu, Wei-Hung Hsu, Shu-Ming Tsao, Ai-Jung Tseng, Zhi-Hu Lin, Wei-Jyun Hua, Hsin Yeh, Tzu-En Lin, Chien-Chang Chen, Li-Sheng Chen, Tung-Yi Lin

**Affiliations:** 1 Institute of Traditional Medicine, National Yang Ming Chiao Tung University, Taipei 11221, Taiwan; qgdfms@gm.ym.edu.tw (W.-L.Q.); seanhsu5817@gmail.com (W.-H.H.); aurora.tseng@gmail.com (A.-J.T.); tiger77749@gmail.com (Z.-H.L.); karta603@gm.ym.edu.tw (W.-J.H.); shin28jenna@gmail.com (H.Y.); 2 LO-Sheng Hospital Ministry of Health and Welfare, New Taipei 242, Taiwan; 3 School of Oral Hygiene, College of Oral Medicine, Taipei Medical University, Taipei 110, Taiwan; 4 Department of Biotechnology and Laboratory Science in Medicine, National Yang Ming Chiao Tung University, Taipei 11221, Taiwan; tsaosm@gmail.com; 5 Program in Molecular Medicine, National Yang Ming Chiao Tung University, Taipei 11221, Taiwan; 6 Institute of Biomedical Engineering, National Yang Ming Chiao Tung University, Hsinchu 30010, Taiwan; telin@nctu.edu.tw; 7 The General Education Center, Ming Chi University of Technology, New Taipei 243, Taiwan; chang@mail.mcut.edu.tw; 8 Biomedical Industry Ph.D. Program, National Yang Ming Chiao Tung University, Taipei 11221, Taiwan

**Keywords:** *Ganoderma lucidum*, polysaccharides, cisplatin, synergistic effect, anti-lung cancer

## Abstract

Lung cancer has the highest global mortality rate of any cancer. Although targeted therapeutic drugs are commercially available, the common drug resistance and insensitivity to cisplatin-based chemotherapy, a common clinical treatment for lung cancer, have prompted active research on alternative lung cancer therapies and methods for mitigating cisplatin-related complications. In this study, we investigated the effect of WSG, a glucose-rich, water soluble polysaccharide derived from *Ganoderma lucidum*, on cisplatin-based treatment for lung cancer. Murine Lewis lung carcinoma (LLC1) cells were injected into C57BL/6 mice subcutaneously and through the tail vein. The combined administration of WSG and cisplatin effectively inhibited tumor growth and the formation of metastatic nodules in the lung tissue of the mice. Moreover, WSG increased the survival rate of mice receiving cisplatin. Co-treatment with WSG and cisplatin induced a synergistic inhibitory effect on the growth of lung cancer cells, enhancing the apoptotic responses mediated by cisplatin. WSG also reduced the cytotoxic effect of cisplatin in both macrophages and normal lung fibroblasts. Our findings suggest that WSG can increase the therapeutic effectiveness of cisplatin. In clinical settings, WSG may be used as an adjuvant or supplementary agent.

## 1. Introduction

*Ganoderma lucidum* is a well-known ingredient in traditional Chinese medicine. *G. lucidum* has long been used in Asian countries, in particular in China, where it is regarded as an elixir that promotes health and longevity [1,2,3]. Modern pharmaceutical studies have reported various biological properties of *G. lucidum* (e.g., anticancer, antioxidant, antimicrobial, immunomodulatory, and anti-inflammatory) [4,5,6,7,8]. The three main components of *G. lucidum*, polysaccharides, triterpenoids, and proteins, have been extensively examined [9,10]. Notably, *G. lucidum* polysaccharides (GLPs) have been commercially tested as clinical drugs and supplements for the treatment of several inflammatory diseases because of the anti-inflammatory, antioxidant, and immunomodulatory activities [11,12]. In addition, one study effectively used GLPs as a material for nanoparticle conjugation because they mediated immunocyte activation and increased radio-sensitivity in the tumor microenvironment [13].

In the past several decades, numerous studies have demonstrated that GLPs play a pivotal role in immunomodulatory and anticancer activities. For example, in one study, GLPs-RF3, a kind of GLP, activates toll-like receptor 4 (TLR4)-mediated extracellular signal-regulated kinase (ERK), c-Jun N-terminal kinase, and p38, leading to induce interleukin-1 expression in mouse spleen cells [14]. GLPs induce various types of cytokine expression to activate macrophages and lymphocytes [6] and accelerate the recovery of cyclophosphamide-disrupted immune functions [15]. In a mouse model, β-_D_-glucan from GLPs effectively inhibit sarcoma-180 solid tumors [16]. GLPs-RF3 attenuates breast cancer tumorigenesis by enhancing the ubiquitination-dependent degradation of transforming growth factor beta (TGF-β) receptors [17]. Other investigations of GLPs have focused on the association between their immunocyte-activating and anticancer activities. For example, GLPs exhibit antitumor activities through the TLR4-dependent activation of B cells and macrophages in a BALB/c mouse model [18]. FMS, a fucose-enriched polysaccharide purified from GLPs-RF3, suppresses lung tumorigenesis through the induction of anti-GloboH-series epitopes antibodies [5]. Taken together, these studies indicate that treatment with GLPs can effectively suppress tumor growth by enhancing the activities of tumor-associated immune effector cells, suggesting that GLPs may constitute an efficacious immunopotentiating adjuvant to anticancer drugs.

Cisplatin, a platinum-based agent, is a well-known anticancer drug that has long been used as an effective clinical chemotherapeutic agent [19]. Its interference with DNA repair mechanisms leads to DNA damage and subsequently triggers apoptotic responses [20]. Clinically, cisplatin has advantages in survival and symptom control in lung cancer therapy because many patients are usually diagnosed with locally advanced or metastatic disease when they are diagnosed with lung cancer. Therefore, cisplatin and its derivatives constitute the first-line drugs for clinical chemotherapy of lung cancer. However, patients undergoing cisplatin-based therapy often experience side effects, such as immunosuppression [21,22]. Studies have reported that combination treatments of cisplatin and herbal medicines exhibited greater efficacy than cisplatin alone did [23,24]. These studies suggest that herbal extracts combined with cisplatin may be a novel cancer treatment strategy. Currently, increasing studies have indicated that GLPs could be considered for use as supplements for patients with cancer [25]. However, the efficacy of combination treatment with GLPs and chemotherapy for lung cancer has yet to be established. Therefore, the synergistic effects of GLPs and cisplatin in lung cancer therapy must be explored.

We previously identified the chemical characterization of WSG, a water soluble glucose-rich polysaccharide derived from GLPs-RF3, and demonstrated it presents anti-lung cancer activity via downregulation of EGFR and TGFβR [26]. In this study, we aimed to investigate whether WSG in combination with cisplatin could overcome lung cancer progression *in vitro* and *in vivo*. Furthermore, we assessed the benefits of co-treatment with WSG and cisplatin on cell viability of macrophages and lung fibroblast *in vitro*.

## 2. Materials and Methods

### 2.1. Materials and Antibodies

WSG, a water soluble glucose-rich polysaccharide with an average molecular mass of approximately 1000 kDa, was isolated from GLPs-RF3 (Wynlife Healthcare, Inc., San Diego, CA, USA. Cat: 500T; Lot: WT20010703) as described previously [26]. Cisplatin (P4394) was purchased from Sigma-Aldrich (Saint Louis, MO, USA). An Annexin V/PI apoptosis kit was purchased from Life Technologies (Carlsbad, CA, USA). Anti-β-actin and anti-EGFR antibodies were acquired from GeneTex (Hsinchu, Taiwan). Anti-phosphorylated AKT (S473) antibody was purchased from Santa Cruz Biotechnology (Santa Cruz, CA, USA), anti-phosphorylated ERK1/2 antibody was purchased from Sigma-Aldrich (Steinheim, Germany).

### 2.2. Mouse Allogeneic Tumor Model

Six-week-old C57BL/6 male mice were purchased from the National Laboratory Animal Center (Taipei, Taiwan) and kept in specific pathogen-free conditions in the Laboratory Animal Center of National Yang Ming Chao Tung University. The experimental procedures and study design were approved by the Institutional Animal Care and Use Committee (approval No: 1090215). After one week of adaptation, the mice were randomly assigned to one of four groups, with five individuals in each group. In the control group, the mice were intraperitoneally injected with phosphate-buffered saline (PBS). In the WSG group, the mice were intraperitoneally injected with 75 mg/kg WSG every 2 days [5,26]. In the cisplatin group, the mice daily received intraperitoneal injections of 2.3 mg/kg cisplatin from day 1 to day 5. In the combination treatment group, the mice were co-treated with WSG and cisplatin.

To create a tumor growth mouse model, LLC1 cells (2 × 10^5^ cells in 100 μL of serum-free medium) were subcutaneously injected into the right flank. Tumors were calculated at 2-day intervals with digital vernier calipers, and tumor volume was calculated as follows: *V =* (*L* × *W*^2^)/2, where *V* is volume in cubic millimeters, *L* is tumor length in millimeters, and *W* is tumor width in millimeters [27]. The mice were sacrificed using carbon dioxide (CO_2_) on day 21. To create a tumor homing mouse model, LLC1 cells (2 × 10^5^ cells in 100 μL of serum-free medium) were injected into the lateral tail vein. The mice were sacrificed using CO_2_ after 28 days. The lung tissue of each mouse was harvested and fixed in paraformaldehyde (10%) for the examination of tumor homing and growth. Hematoxylin and eosin (H&E) staining was applied to the tumor colonies, and the number and tumor volume of the colonies were determined through microscopy.

### 2.3. Cell Lines and Cell Culture

Human lung adenocarcinoma A549 cells, murine LLC1 cells, human lung fibroblast MRC-5, and murine macrophages Raw264.7 were acquired from the Bioresource Collection and Research Center (BCRC; Hsinchu, Taiwan). Human lung adenocarcinoma CL1-5 cells were obtained from Dr. P.-C. Yang of National Taiwan University. The lung cancer cell lines were cultured as previously described [28,29]. The MRC-5 cells were cultured in minimum essential medium supplemented with 10% heat-inactivated fetal bovine serum (FBS; VWR Life Science Seradigm, Radnor, PA, USA), 2 mM L-glutamine, 0.1 mM nonessential amino acids, 1.5 g/L sodium bicarbonate, and 1.0 mM sodium pyruvate at 37 °C in an environment of 95% air and 5% CO_2_. The RAW 264.7 cells were kept in Dulbecco’s modified Eagle medium (GIBCO-Life Technologies) that was supplemented with 3.7 g/L NaHCO_3_, 5% heat-inactivated FBS, and 100 units/mL each of penicillin and streptomycin (Biological Industries, Cromwell, CT, USA).

### 2.4. Cell Viability and Synergistic Analysis

Cell viability was evaluated using a crystal violet assay. For each experiment, 5 × 10^4^ cells were seeded into each well of 12-well plates. After incubation overnight to allow for cell attachment, the cells were subjected to treatment with various concentrations of WSG or cisplatin or a combination of the two for 24 or 48 h. Subsequently, the cells were rinsed with PBS and stained with 1% crystal violet solution for 1 h. The specifics of the procedure were described previously [23]. The percentages of cell viability were normalized for comparison with the control group. Next, as conducted in our previous study [23], the synergistic effects of the combination treatment, as indicated by Chou-Talalay combination indexes (CIs), were analyzed using the CompuSyn program (Paramus, NJ, USA) [30]. The CI was <1, confirming the synergistic effects between the tested drugs.

### 2.5. Cell Protein Extraction for Western Blotting

The cancer cells were rinsed two times with 1 mL of cold PBS supplemented with 1% Na_3_VO_4_ and then scraped into a lysis buffer containing a mixture of proteinase (Sigma-Aldrich, Saint Louis, MO, USA) and phosphatase inhibitors (MedChem Express, Monmouth Junction, NJ, USA) [31]. Next, the cell lysates were centrifuged at 4 °C and 13,000× *g* for 10 min to obtain the supernatant as the cellular proteins or extracts. Protein concentrations were determined using a Bradford assay (Bio-Rad, Hercules, CA, USA). For Western blotting, 30 μg cell extracts were separated through 10% sodium dodecyl sulfate–polyacrylamide gel electrophoresis (SDS-PAGE) and transferred to polyvinylidene difluoride (PVDF) membranes to detect the indicated molecules, with β-actin used as the loading control. The Western blotting assay was performed as described previously [32].

### 2.6. Sample Preparation for Apoptosis Analysis

The cancer cells were seeded in 6-cm plates at a density of 2 × 10^5^ cells/plate. After overnight incubation, the cells were treated with WSG (120 μg/mL) and/or cisplatin (10 μM) for 48 h and subsequently washed with PBS and harvested after trypsinization. The cell pellets were washed with cold PBS, centrifuged, and then collected. Next, the cell pellets were re-suspended in annexin-binding buffer and stained with propidium iodide (PI) and annexin V–fluorescein isothiocyanate (FITC) using an apoptosis detection kit (BD Pharmingen, USA) in accordance with the manufacturer’s instructions. The samples were examined using a flow cytometer (BD FACSCalibur, Franklin lakes, NJ, USA), and the raw data were analyzed for apoptosis by using FlowJo software (National Institute of Mental Health, Bethesda, MD, USA).

### 2.7. Statistical Analysis

All presented results are representative of three independent experiments unless otherwise indicated. All data are expressed as means ± standard deviations of the indicated number of experiments. Between-group differences were analyzed through a *t* test using GraphPad Prism8 software (San Diego, CA, USA). Differences were considered significant at *p* < 0.05.

## 3. Results and Discussion

### 3.1. WSG Synergistically Enhances Cisplatin-Induced Cytotoxicity in Lung Cancer Cells

**Figure 1:**
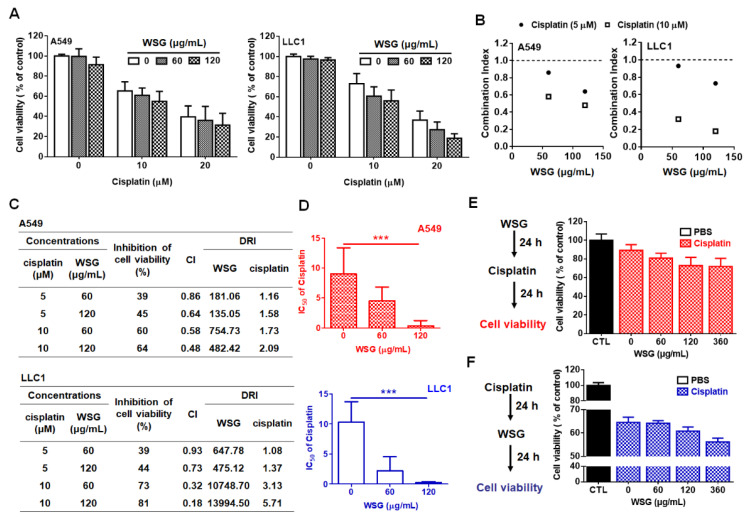
WSG enhances the cytotoxic effects of cisplatin on A549 and LLC1 cells. (**A**) A549 and LLC1 cells were subjected to 24 h combination treatment using different concentrations of WSG and cisplatin. A crystal violet staining assay was conducted for cell viability examination. Each sample in the combination treatment group was normalized against each untreated control. (**B**,**C**) CI and DRI of the combination treatment in the A549 and LLC1 cells, determined using CompuSyn software. (**D**) WSG reduced the IC_50_ of cisplatin in A549 and LLC1 cells. The IC_50_ values of cisplatin were measured using CompuSyn Software. Significant differences between the treatment and control groups are presented (*** *p* < 0.001). (**E**) Left: the schematic design for the in vitro cell viability assay of WSG (0–360 μg/mL)→cisplatin (5 μM) sequential treatment. Right: The viability of the cells was determined by using the crystal violet assay. (**F**) Left: the schematic design for the in vitro cell viability assay of cisplatin (5 μM)→WSG (0–360 μg/mL) sequential treatment. Right: The viability of the cells was determined by using the crystal violet assay. Each sample in the combination treatment group was normalized against each untreated control. Error bars indicated SD.

Previously, we have demonstrated that WSG effectively inhibited the viability of A549 and LLC1 cells [26]. Herein, we investigated the impact of WSG combined with cisplatin treatment on the viability of lung cancer cells *in vitro*. A crystal violet assay was performed to examine the efficacy of the combination treatment in suppressing lung cancer A549 and LLC1 cells. The cells were co-treated with cisplatin (0–20 μM) and low-dose WSG (60 or 120 μg/mL) for 24 h. The combination treatment exhibited stronger inhibitory effects than WSG or cisplatin treatment alone (Figure 1A). The interactions between WSG and cisplatin were evaluated with Chou-Talalay CIs [33]. The CIs for all concentrations were less than 1 (Figure 1B), suggesting that the combination treatment exerted a reliable, synergistic, cytotoxic effect on the A549 and LLC1 cells. Regarding the dose-reduction indexes (DRIs) of the combination treatment [33], the DRIs exceeded 1 for both WSG and cisplatin (Figure 1C). Moreover, the IC_50_ of cisplatin in combination with WSG were calculated. As shown in Figure 1D, WSG dramatically decreased the IC_50_ values of cisplatin in A549 and LLC1 cells in a concentration-dependent manner, with WSG of 120 μg/mL reducing significantly the IC_50_ of cisplatin from 10 to 1 μM and 0.5 μM, respectively. These results indicate that the combination of WSG and cisplatin may promote anticancer activity and attenuate the side effects of the individual treatments, supporting a previous finding that GLPs may reduce the occurrence of chemotherapy side effects in patients with cancer [34]. In short, WSG and cisplatin exhibited synergistic cytotoxic effects in the lung cancer cells.

To mimic the potential clinical complementary strategy, we further examined the effect of a sequential treatment of WSG and cisplatin on lung cancer A549 cells. Two types of sequential treatments were performed. Type one (type 1): cancer cells were treated with WSG, followed by cisplatin incubation (Figure 1E; WSG→Cisplatin). By contrast, in the treatment of type two (type 2), cells were treated with cisplatin, followed by WSG incubation (Figure 1F; Cisplatin→WSG). As expected, we found that no matter whether WSG or cisplatin was given first, the sequential treatment presented a more effective inhibition rate of the cell viability (Figure 1E,F). Together, we demonstrated that WSG could enhance the cisplatin-inhibited viability of lung cancer cells *in vitro*.

### 3.2. WSG Enhances Cisplatin-Induced Apoptotic Response in Lung Cancer Cells

**Figure 2:**
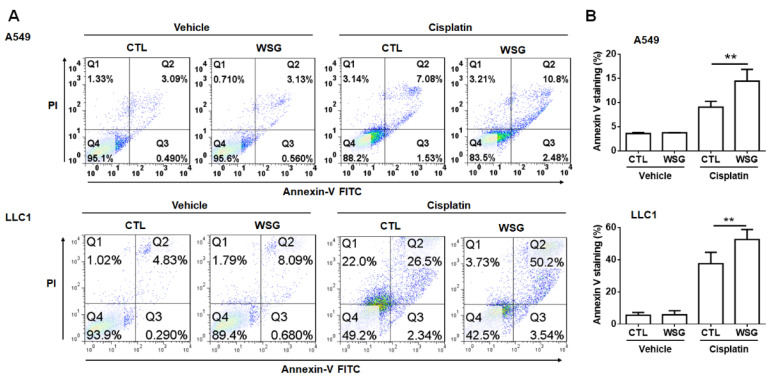
WSG enhances cisplatin-induced apoptotic responses. (**A**) After 48 h exposure to WSG (120 μg/mL) and/or cisplatin (10 μM), the cells were subjected to co-staining with the annexin V–FITC/PI kit. Flow cytometry was performed for apoptosis analysis. The percentages of apoptotic cells in early and late apoptosis were determined using FlowJo software. (**B**) The data, representative of three separate experiments, are presented as means ± standard deviations; error bars reflect standard deviations. Significant differences between the treatment and control groups are presented (** *p* < 0.01).

To further examine these synergistic cytotoxic effects, we used an annexin V–FITC/PI staining kit to test whether WSG enhanced cisplatin-induced cell death. As shown in Figure 2, apoptosis occurred in 32.7–45.7% of the A549 cells co-treated with cisplatin (10 μM) and WSG (120 μg/mL). The corresponding rate among the LLC1 cells ranged from 27.6% to 51.8%. These results suggest that the combination of cisplatin and WSG was much more effective in inducing cell death than cisplatin alone. In essence, the *in vitro* experiments demonstrated that the combination of cisplatin and WSG exerted reliable, synergistic, and cytotoxic effects, inducing cell death in the lung cancer cells.

### 3.3. WSG Retains Viability in RAW 264.7 and MRC-5 Cells Treated with Cisplatin

**Figure 3:**
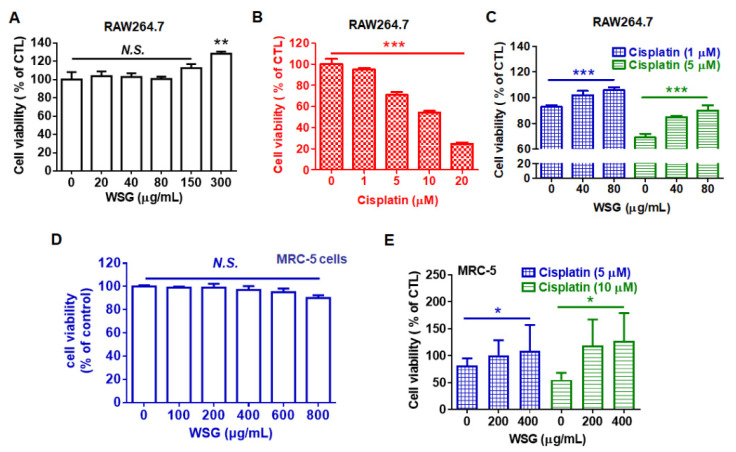
WSG reduces cisplatin-induced cytotoxic effects on RAW 264.7 and MRC-5 cells. (**A**) RAW 264.7 murine macrophages were subjected to 24 h treatment with various concentrations (0–300 μg/mL) of WSG. (**B**) RAW 264.7 cells were subjected to treatment with various concentrations (0–20 μM) of cisplatin for 24 h. (**C**) RAW 264.7 cells were subjected to 24 h treatment with both WSG (40 and 80 μg/mL) and cisplatin (1 and 5 μM). (**D**) MCR-5 cells were subjected to 48 h treatment with various concentrations (0–800 μg/mL) of WSG, and their viability was assessed through crystal violet staining. WSG treatment group data were normalized for comparison with an untreated control. (**E**) MRC-5 fibroblasts were subjected to 48 h treatment with a combination of WSG (200 and 400 μg/mL) and cisplatin (5 and 10 μM). A crystal violet assay was performed for cell viability examination. Treatment groups were normalized for comparison with an untreated control. Data are presented as means ± standard deviations. Significant differences between the treatment and control groups are presented (* *p* < 0.05, ** *p* < 0.01, *** *p* < 0.001).

Patients receiving cisplatin-based therapy often experience complications, including low blood cell counts, irreversible hearing loss (ototoxicity), and permanent neuronal and renal damage [21,22]. According to cisplatin manufacturers, the medication reduces the synthesis of blood cells, specifically white blood cells, leaving patients susceptible to infection. Therefore, we examined the effects of WSG on cisplatin-treated RAW 264.7 macrophages. Low concentrations of WSG (20–150 μg/mL) did not affect viability, but a high concentration (300 μg/mL) increased viability by approximately 30% (Figure 3A). We also examined the cytotoxic effect of cisplatin on RAW264.7 cells. As expected, cisplatin substantially and dose-dependently reduced the viability of the RAW 264.7 cells (Figure 3B), consistent with results of a previous study [35]. Notably, WSG increased the viability of RAW 264.7 cells upon cisplatin treatment (Figure 3C). Previous study shows that polysaccharides from *Cudrania tricuspidata* fruit mediated with a reduction in reactive oxygen species (ROS) production and mitochondrial transmembrane potential loss contributes to cytoprotective action in cisplatin-treated RAW264.7 cells [36]. Whether WSG could mediate cisplatin-induced ROS production and mitochondria damage needs to be dissected in the future. In addition, cisplatin exerted cytotoxic effects on the MRC-5 cells, in line with previous results [37]. We found that WSG did not exhibit cytotoxic effects on the MCR-5 cells (Figure 3D) but increased cell viability of the MRC-5 cells upon cisplatin treatment (Figure 3E). Increasing evidence shows that GLPs might attenuate the cytotoxicity induced by cisplatin to improve chemotherapy-induced fatigue [38]. Herein, we demonstrated that WSG protects white blood cells and lung fibroblasts from the adverse side effects of cisplatin. The results collectively indicate that WSG may be a safe anticancer agent for inhibiting the survival of lung cancer cells.

### 3.4. Co-Treatment with WSG and Cisplatin Effectively Suppresses Lung Tumor Growth in LLC1-Bearing Mice

**Figure 4:**
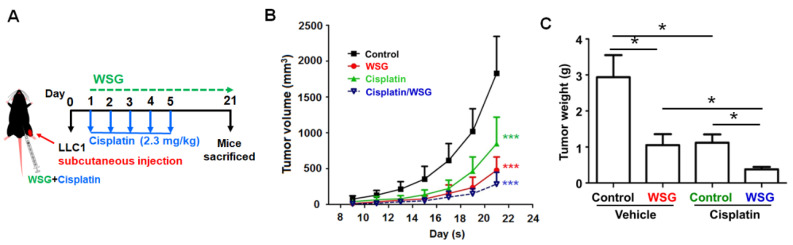
Co-treatment with WSG and cisplatin suppresses lung tumor growth in mice bearing LLC1 cells. (**A**) Experimental schematic. LLC1-bearing mice (*n* = 5 in each group) were intraperitoneally injected with cisplatin and WSG. (**B**) Tumor volume was monitored for the indicated times. (**C**) Tumor weights were determined after each intervention. The bars represent means ± standard deviations. Significant differences between the treatment and control groups are presented (* *p* < 0.05, *** *p* < 0.001).

As the above results show, co-treatment with WSG and cisplatin effectively inhibits lung cancer cells *in vitro*. We further explored the impact of WSG co-treatment with cisplatin on lung tumor growth *in vivo*. Initially, LLC1 cells were hypodermically injected into the hypodermic dorsum of mice that were randomly divided into four groups: control (PBS), WSG, cisplatin, and WSG combined with cisplatin. The tumor growth rates were monitored over 21 days (Figure 4A). As expected, compared with the control group, both WSG and cisplatin effectively inhibited tumor growth and reduced tumor weight in the LLC1-bearing mice (Figure 4B,C), and stronger anticancer activity was achieved with WSG than with cisplatin. The tumor-suppressive effects of co-treatment with cisplatin and WSG in the LLC1-bearing mice were significantly stronger than those of WSG or cisplatin alone (Figure 4B,C). In addition, the combination treatment did not affect the body weight of the mice, indicating the nontoxicity of this therapeutic strategy (data not shown). These results suggest that WSG may function both as an antitumor agent and as a chemotherapeutic adjuvant enhancing the anticancer effects of cisplatin.

### 3.5. Combination of WSG and Cisplatin Effectively Suppresses Tumor Growth in Lung Tissues of LLC1 Cells-Bearing Mice

**Figure 5:**
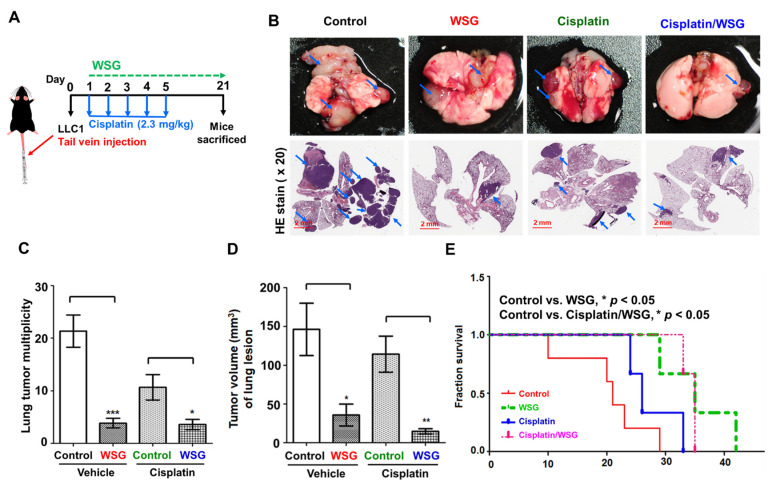
Co-treatment with WSG and cisplatin suppresses tumorigenesis in the lung tissues of mice injected with LLC1 cells. (**A**) Experimental schematic. LLC1 cells were injected through the lateral tail veins of the mice (*n* = 5). The LLC1-bearing mice were intraperitoneally injected with cisplatin and WSG. (**B**) At the end of the treatment, lung tissue was collected. One of five tissue samples is presented. As indicated by the blue arrows, tumor lesions were observed on the surface of the lung tissues. Lower panels of the lung sections under H&E staining (20× magnification). Bar scale: 2 mm. (**C**) Tumors (nodules) in the lung lesions. (**D**) Tumor volume (mm^3^) of the lung lesions. Each bar represents the mean ± standard deviation. Significant differences between the treatment and control groups are presented (* *p* < 0.05, ** *p* < 0.01, *** *p* < 0.001). (**E**) Combination treatment increased the survival rate of LLC1-bearing mice (*n* = 8).

We further investigated the antitumor effects of the combination treatment on the lung tissue of the mice (Figure 5A). To mimic tumor progression in the lung tissue, we injected LLC1 cells into the mice through the lateral tail vein to trap them and allow them to develop into tumor lesions [39]. We initially counted the tumor nodules in the lung tissue of mice receiving the combination treatment. As shown in Figure 5B,C, WSG significantly suppressed lung cancer homing and growth, a result observed in our previous study [26]. Specifically, the combination treatment reduced the number of tumor nodules considerably more than cisplatin treatment alone (Figure 5B,C). H&E staining of the tumor nodules revealed fewer instances of tumor homing (nodules) in the lung tissue of the mice receiving the combination treatment than in the tissue of the mice receiving WSG or cisplatin alone (Figure 5B). However, it did not exert stronger tumor-suppressive effects than WSG treatment did alone (Figure 5C). Notably, the combination treatment reduced tumor volume substantially more than WSG or cisplatin alone (Figure 5D), suggesting that the combination strategy not only inhibited tumor nodules formation but also suppressed tumor growth in lung tissues. In addition, the survival rate of the mice receiving the combination treatment was significantly higher than that of the mice receiving the control treatment or cisplatin alone (Figure 5E). However, the mice treated with WSG alone had the highest overall survival rate. Our results indicate that WSG has potential as a therapeutic intervention for controlling disease progression and extending life in lung cancer.

In this study, our results showed that the combination with WSG potentiated the cell-killing efficacy (apoptosis) of cisplatin in lung cancer cells in vitro. More remarkably, we found that the antitumor effect of cisplatin was significantly enhanced with the combination with WSG in the lung cancer-bearing mouse model. Notably, the combination of WSG with cisplatin showed selectivity in enhancing the efficacy of cisplatin between cancer cells and normal cells, suggesting that WSG may play different roles for contributing to the cellular responses in cisplatin-treated lung cancer and normal cells. Increasing evidence shows that multiple intracellular regulators may contribute to cisplatin resistance [40]. For instance, the excision repair cross-complementation group 1 (ERCC1) enzyme plays a pivotal role in cisplatin-induced DNA damage and recombination. Clinically, patients with lung cancer and ERCC1-positive tumors appear to worsen from adjuvant cisplatin-based chemotherapy [41]. Expression of copper-transporting P-type adenosine triphosphatase A (ATP7A) is associated with cisplatin resistance in lung cancer cells [42]. However, WSG did not modulate the expression of ERCC1 and ATP7A (data not shown), suggesting that WSG may not be involved in the cisplatin-associated DNA repair system and copper-transporting. Notably, ERK signaling contributes to cisplatin resistance [43]. We previously found WSG significantly reduced phosphorylation of ERK1/2 [26], suggesting that WSG improved the activity of cisplatin against lung cancer cells via the targeting of ERK signaling.

## 4. Conclusions

WSG is a novel glucose-rich polysaccharide isolated from commercially available GLPs-RF3. We previously identified the potential anticancer mechanism of WSG [26]. Cisplatin-based chemotherapy is one of the most common and effective treatments for lung cancer [44]. To investigate the clinical applications of WSG, we conducted a preclinical study of WSG on tumor-bearing mice receiving chemotherapy. WSG enhanced the tumor-suppressive effects of cisplatin *in vivo* as well as *in vitro*. In addition, WSG enhanced the cytotoxic and tumor-suppressive effects of cisplatin and increased the survival rates of LLC1-bearing mice. Furthermore, in macrophages and lung fibroblasts, WSG attenuated the cytotoxic effects of cisplatin. Thus, WSG may be considered for use as an adjuvant or dietary supplement to improve outcomes in patients receiving cisplatin-based chemotherapy.

## Data Availability

The data presented in this study are available on request from the corresponding author.
